# Outcomes of Collagen and Conventional Dressings in the Management of Diabetic Foot Ulcers: A Comparative Study in Bangladesh

**DOI:** 10.7759/cureus.94306

**Published:** 2025-10-10

**Authors:** Md Arifur Rahman, Zain Girach, Shahper Reza, Imran Kais, M Samia Islam, Nur Jenny

**Affiliations:** 1 Department of General Surgery, Kettering General Hospital NHS Foundation Trust, Kettering, GBR; 2 Department of General Surgery, Holy Family Red Crescent Medical College and Hospital, Dhaka, BGD; 3 Department of General Surgery, Leicester Royal Infirmary, University Hospitals of Leicester NHS Trust, Leicester, GBR; 4 Department of Oral and Maxillofacial Surgery, Dhaka Dental College and Hospital, Dhaka, BGD; 5 Department of General Surgery, Chesterfield Royal Hospital NHS Foundation Trust, Chesterfield, GBR

**Keywords:** collagen dressing, coventional dressing, diabetic foot ulcer management, diabetic foot ulcers (dfus), diabetic wound healing, granulation time

## Abstract

Introduction: Diabetic foot ulcers (DFU) are a major consequence of diabetes and a major cause of lower-extremity amputation. Effective management techniques are essential given this high burden, especially in Bangladesh. The purpose of this study was to compare the effectiveness of collagen and traditional dressings in the treatment of DFU patients.

Methods: An observational comparative cross-sectional study was conducted in the Department of Surgery, Dhaka Medical College Hospital, Bangladesh, over six months. A total of 100 patients with DFU were enrolled and equally divided into two groups: one treated with collagen dressing and the other with conventional dressing. Patients were recruited following inclusion and exclusion criteria, and informed consent was obtained. Data on socio-demographic characteristics, glycemic control, and treatment outcomes were collected. Outcome assessment focused on time to granulation and complete healing. Data were analyzed using Stata Statistical Software: Release 13 (StataCorp LLC, College Station, Texas, United States).

Results: The mean age of participants was 60.07±10.39 years (range: 34-78 years); 77% (n=77) were male. Mean HbA1c was comparable between groups (collagen: 8.12±1.14% vs. conventional: 8.05±0.74%). Granulation occurred significantly earlier in the collagen group (2.26±1.58 weeks) than in the conventional group (3.76±1.57 weeks; p<0.001). Similarly, mean healing time was shorter with collagen (4.90±2.54 weeks) compared to conventional dressing (6.24±3.76 weeks; p<0.05). Hazard ratio (HR) analysis showed granulation tissue appeared 1.96 times more likely with collagen dressing than conventional (HR: 1.96; 95%CI: 1.31-2.96; p<0.05).

Conclusion: DFUs, which involve prolonged healing and a high risk of infection, are a major cause of morbidity, mortality, and hospitalization in diabetic patients. This study found that collagen dressing outperforms conventional dressings, promoting faster healing and earlier granulation, with granulation tissue nearly twice as likely to appear early.

## Introduction

Diabetes mellitus (DM) is a common worldwide problem affecting 537 million people, according to the International Diabetes Federation, with 7.1 million people suffering from diabetes in Bangladesh. Diabetic foot ulcer (DFU) is one of the most complex and heterogeneous complications in patients with diabetes mellitus [1]. 

DFUs, along with the associated illness and death resulting from foot complications in individuals with diabetes, pose a significant public health concern [2]. Severe foot and lower limb issues are the leading reasons for hospital admissions among diabetic patients. In addition, between 40% and 70% of all major non-trauma-related lower limb amputations occur in people with diabetes [3]. DFU is also associated with the disruption of normal wound healing mechanism. The persistent inflammation in DFU is likely due to bacterial contamination and subsequent infections [4].

A topical intervention method like dressing has been used for the treatment of DFU for quite a long time, and in Bangladesh, conventional dressing is the most commonly used dressing in the case of this ulcer. However, limited healthcare resources and restricted access to advanced wound care products like collagen dressings pose significant challenges in many regions of Bangladesh.

Wound healing is a multifaceted process that relies on the coordinated release of various growth factors to support cell movement and growth, the formation of new connective tissue, and the deposition of collagen [5,6]. However, DFUs represent a type of chronic wound that remains stalled in the inflammatory phase and fails to support the proper regeneration or migration of epidermal cells across the wound site [7,8]. One hallmark of chronic wounds is the increased presence of matrix metalloproteinases (MMPs), which elevate proteolytic activity and degrade the growth factors necessary for healing. Collagen-based treatments are believed to selectively inhibit these proteases without interfering with the beneficial effects of growth factors. This suggests that collagen could potentially offer a more effective treatment option compared to traditional moist gauze, which is currently the standard approach.

New advanced topical dressings are emerging that may improve wound care. Such dressings are designed to modulate levels of biological molecules, such as growth factors, that may promote wound healing. Among newer types of wound dressings, biological dressings like collagen create the most physiological interface between the wound surface, environment and are impermeable to bacteria [3]. Collagen is a key structural protein found in connective tissue, and increasing research is shedding light on its biochemical properties and its important role in the wound healing process [4-7].

Several studies have explored the effect of collagen dressing in wound healing. Some studies have found collagen dressing to be safe and effective in the treatment of foot ulcers and significantly reduce healing time, duration of antibiotic therapy, and follow-up time [8-11]. Furthermore, several studies have reported the superior efficacy of collagen dressing over conventional dressing for foot ulcers [11,12].

DFU is not uncommon in a tertiary-level hospital like the Dhaka Medical College Hospital of Bangladesh, and both conventional and collagen dressings are being used in the management of DFUs. However, no study has been conducted in this field to compare these two dressing materials in Bangladesh. Thus, this study aimed to create necessary evidence-based knowledge with regard to the management of DFU in the context of Bangladesh. The objective of this study was to compare the effectiveness of collagen dressings versus conventional dressings in promoting healing in DFUs, with a focus on granulation tissue formation and overall healing time. This was achieved by measuring the time of appearance of healthy granulation tissue in patients with DFU using conventional and collagen dressings, and by comparing the healing time after using the two dressings. Thus, this will create scientific evidence that may ease the choice of dressing material for DFUs.

## Materials and methods

An observational, comparative, cross-sectional study was carried out in the Department of Surgery, Dhaka Medical College Hospital, Bangladesh, for a total of six months. The study population involved 100 patients with DFU. The research protocol was approved by the Ethical Review Committee of Dhaka Medical College (approval number: MEU-DMC/ECC/2017/67). Informed consent was obtained from all participants after explaining the study’s objectives and purpose. Participants were assured of their right to withdraw from the study at any time without any consequences. They were also informed that their personal information would remain confidential. Additionally, it was made clear that their participation would not influence their medical treatment or care in any way, nor would they receive any financial compensation.

Selection criteria

The inclusion criteria included adult patients with DFU who were over the age of 18 and were willing to participate in the study. The exclusion criteria were patients with a malignancy, patients with underlying osteomyelitis, and seriously ill patients. Patients who did not provide consent or whose data were incomplete were also excluded from the study. All patients who were admitted for DFU during the study period and fulfilled the inclusion/exclusion criteria were selected for the study.

Operational definitions

This study consisted of two main measurements: the ulcer healing time and the time to appearance of granulation tissue. The ulcer healing time was defined as the time required to complete healing of the ulcer after the initiation of the therapy with collagen/conventional dressings in patients with DFU. The appearance of granulation tissue was defined as the time required for the formation of granulation tissue after the start of therapy with collagen and conventional dressing in patients with DFU.

Study procedure

Patients admitted to the Department of Surgery for the treatment of DFU were included as the study population. Upon admission, each patient underwent a clinical evaluation and diagnostic investigations to confirm the diagnosis and identify any existing comorbid conditions. 

After initial resuscitation and stabilization, all patients received care based on the standard treatment protocol. Participants were then randomly assigned to one of two groups using a lottery method. One group (n=50, 50%) received conventional wound dressing, while the other group (n=50, 50%) was treated with collagen dressings. Dressings were applied by the investigator, ensuring consistent use of standardized procedures across both groups. Conventional wound dressings were changed at least once every other day, and collagen wound dressings at least once every seven days. However, due to multiple factors, such as the amount of exudate, the presence of infection, and the patient's overall condition, this varied. 

Data collection

Data were collected consecutively throughout the study period. A structured questionnaire (see Appendices) was used to collect patient information, including sociodemographic details and relevant clinical data. 

Data processing and analysis

After the collection of all the required data, it was verified for consistency and then tabulated into the computer using Microsoft Excel (Microsoft Corporation, Redmond, Washington, United States). Data were analyzed using Stata Statistical Software: Release 13 (StataCorp LLC, College Station, Texas, United States).

Statistical significance was considered at a 95% confidence level with 5% acceptable error level. To compare the demographic data of patients with DFU with conventional dressing and collagen dressing, descriptive statistics were used. Patients' characteristics were reported as percentages or mean ± standard deviation (SD). To compare the difference between granulation time and healing time of study subjects between groups, the chi-square test was used. Moreover, a univariate Cox regression analysis was done to estimate the effect of collagen dressing in the formation of granulation tissue as well as in the healing and expressed as hazard ratio (HR). Differences were considered significant at the p < 0.05 level for all these tests.

## Results

**Figure 1:**
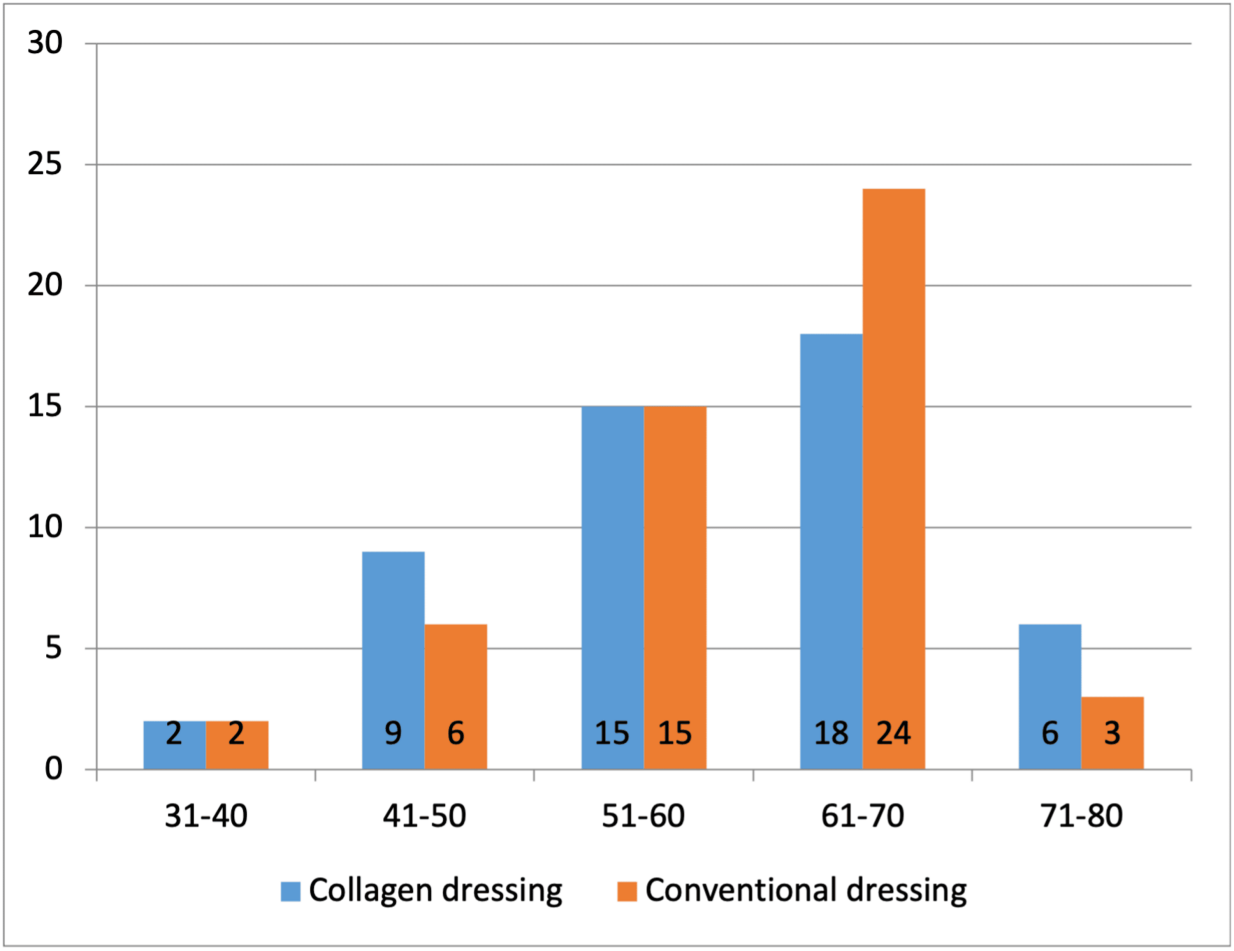
Age group distribution of the subjects (N=100)

A total of 100 patients with DFU were included in this study. Patients were divided into two groups for treatment. One group was treated with conventional dressings (n=50), and the other group was treated with collagen dressings (n=50). The mean age of the total participants was 60.07±10.39 years, with ages ranging from 34 years to 78 years. The mean age of the collagen dressing group was 59.66±10.72 years, and of the conventional dressing group was 60.48±10.14 years. The difference was not significant (p>0.05). The majority of the patients (42%, n=42) were aged 61-70 years (Figure 1). 

**Figure 2:**
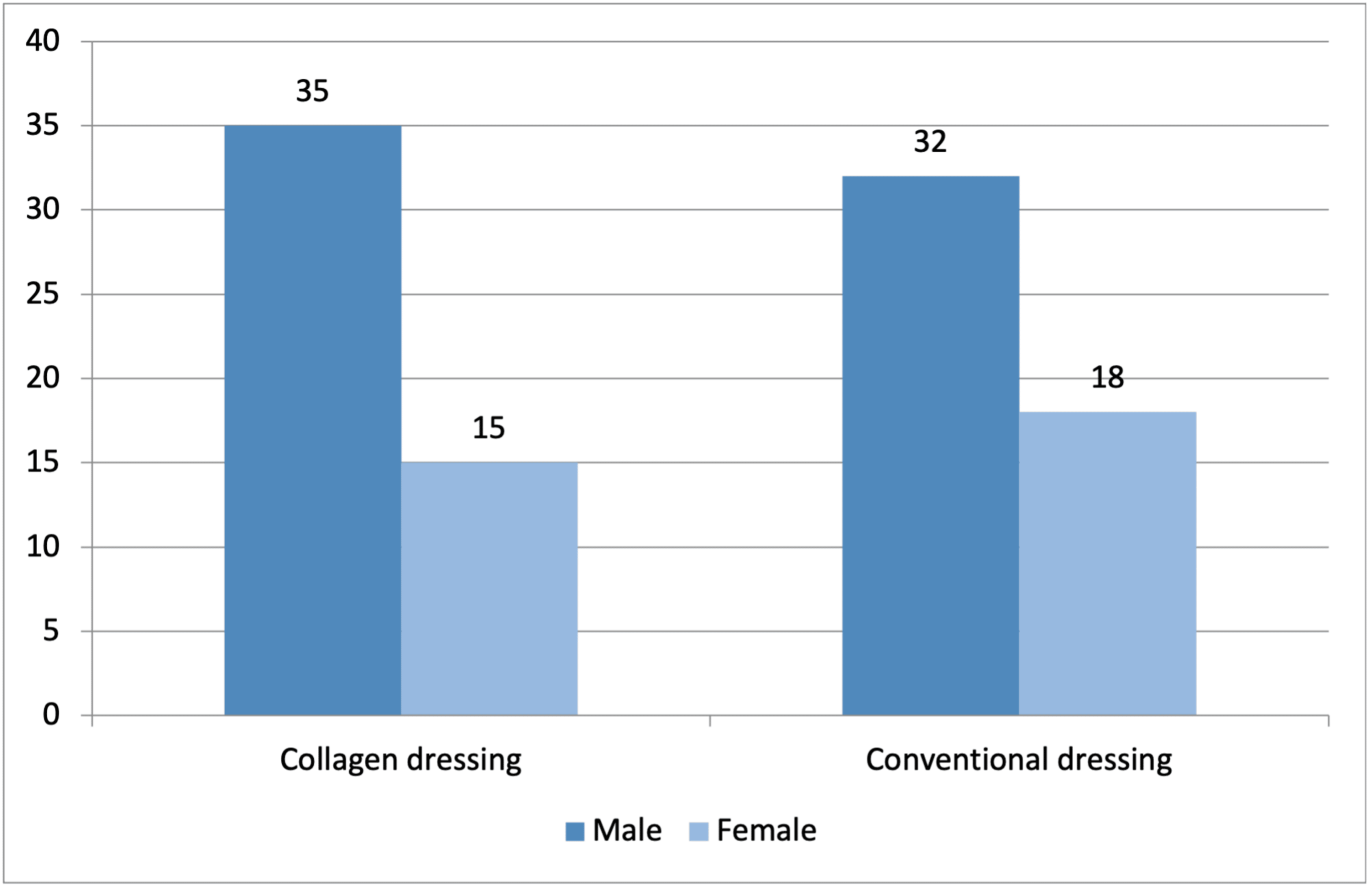
Distribution of subjects according to sex (n=100)

Among 100 subjects, 77% (n=77) were male and 33% (n=33) were female. In the collagen dressing group, 70% (n=35) were male and 30% (n=15) were female, while in the conventional dressing group, 64% (n=32) were male and 36% (n=18) were female (Figure 2).

Of the study participants, 96% (n=96) were followers of Islam. With regard to residence, a slightly higher proportion of participants came from rural areas (52%, n=52). Among all participants, 21% (n=21) were housewives, 19% (n=19) were doing business, 15% (n=15) were doing government service, 15% (n=15) were doing non-government service, and 30% (n=30) were doing other jobs such as rickshaw puller, van driver, farmer, and day laborer (Table 1).

**Table 1:** Socioeconomic profile of study participants (N=100)

Variable	Collagen dressing (n=50), n (%)	Conventional dressing (n=50), n (%)	Total, n (%)
Religion			
Islam	48 (96)	48 (96)	96 (96)
Hindu	2 (4)	2 (4)	4 (4)
Residence			
Rural	27 (54)	25 (50)	52 (52)
Urban	23 (46)	25 (50)	48 (48)
Occupation			
Government Service	6 (12)	9 (18)	15 (15)
Non-government service	7 (14)	8 (16)	15 (15)
Business	11 (22)	8 (16)	19 (19)
Housewife	11 (22)	10 (20)	21 (21)
Others	15 (30)	15 (30)	30 (30)

**Figure 3:**
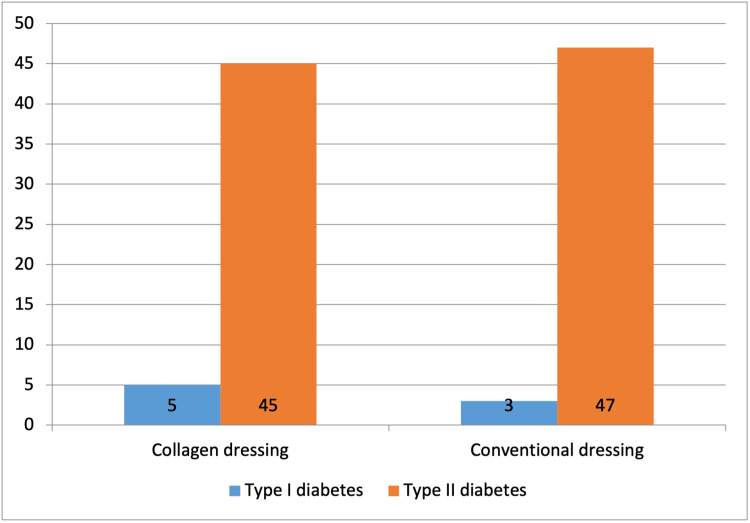
Distribution of participants according to type of diabetes (N=100)

A total of 92 (92%) participants had type II diabetes mellitus, and 8% (n=8) had type I diabetes mellitus. The distribution of the type of diabetes was similar across groups (Figure 3).

The mean HbA1C of the study participants was 8.09±0.96. It was 8.12±1.14 and 8.05±0.74 for the collagen dressing and the conventional dressing group, respectively. Neuropathy (chi-square value 0.06 and p value 0.81) was present in 78% (n=78) of patients, and arterial insufficiency was present in 7% (n=7) of patients (Table 2).

**Table 2:** Clinical and biochemical profile of study participants (N=100)

Variable	Collagen dressing (n=50)	Conventional dressing (n=50)	Total	Chi-square values	P value
HbA_1_C, mean±SD	8.12±1.14	8.05±0.74	8.09±0.96	-	-
Neuropathy, n				0.06	0.81
Yes	40	38	78		
No	10	12	22		
Arterial Insufficiency, n				0.00	1.00
Yes	4	3	7		
No	46	47	93		

The duration of ulcer in the patients ranged from one to 82 months, with a median of four months. Mean wound area of the collagen dressing group was 5.91 cm^2 ^(range 0.57-17.80), and that of the conventional dressing group was 5.15 cm^2^ (range 0.40-18.40). The majority of the wounds were found in the plantar surface of the foot (73%, n=73). Serous discharge was a common finding in these wounds (70%, n=70) (Table 3).

**Table 3:** Characteristics of ulcers of study participants (N=100)

Variable	Collagen dressing (n=50)	Conventional dressing (n=50)	Total	Chi-square value	P value
_­_Duration (months), median (range)	4 (1-72)	4 (1-82)	4 (1-82)	-	-
Wound area (cm^2^), mean (range)	5.91 (0.57-17.80)	5.15 (0.40 – 18.40)	5.53 (0.40 – 18.40)	-	-
Site, n (%)				0.96	0.81
Plantar Surface of foot	36 (72)	37 (74)	73 (73)		
Back of heel	4 (8)	6 (12)	10 (10)		
Tip of toes	4 (8)	3 (6)	7 (7)		
Dorsum of foot	6 (12)	4 (8)	10 (10)		
Discharge, n (%)				1.28	0.53
Serous	37 (74)	33 (66)	70 (70)		
Purulent	3 (6)	6 (12)	9 (9)		
None	10 (20)	11 (22)	21 (21)		

Granulation time occurred in significantly shorter duration in the collagen dressing group(2.26±1.58 weeks) in comparison to the conventional dressing group (3.76±1.57 weeks, p<0.001). Also, healing time was significantly lower in the collagen dressing group (4.90±2.54 weeks) than in the conventional dressing group (6.24±3.76 weeks, p<0.05) (Table 4).

**Table 4:** Granulation time and healing time of study participants (N=100) * p obtained by independent samples Student’s t test

Variable	Collagen dressing (n=50)	Conventional dressing (n=50)	Chi-square values	P Value*
Granulation time in weeks mean±SD (range)	2.26 ±1.58 (1 – 7)	3.76 ±1.57 (1 – 8)	≥10.8	<0.001
Healing time in weeks mean±SD (range)	4.90 ± 2.54 (1 – 12)	6.24 ±3.76 (2 – 12)	6.7	0.01

A univariate Cox regression analysis was done to estimate the effect of collagen dressing on the formation of granulation tissue as well as on the healing. Patients who were treated with collagen dressing had a significant HR of 1.96 (95%CI 1.31-2.96, p <0.05) for the development of granulation tissue compared to those treated with conventional dressing. Also, the collagen dressing group had a significant HR of 1.53 (95%CI 1.03 - 2.27, p <0.05) for healing compared to the conventional dressing group (Table 5). 

**Table 5:** Hazard ratios (HRs) of granulation and healing in collagen in comparison to conventional dressing (N=100) * p obtained by unvariate Cox regression analysis

Variable	HRs	95% CI	Chi-square values	p Value*
Granulation time				
Conventional dressing	Reference	-	-	-
Collagen dressing	1.96	1.31 – 2.96	10.8	0.001
Healing time				
Conventional dressing	Reference	-	-	-
Collagen dressing	1.53	1.03 – 2.27	4.7	0.03

## Discussion

DFU is a common, complex, and costly complication of diabetes mellitus. According to the International Diabetes Federation prevalence data, foot ulcers develop in 9.1-26.1 million people with diabetes worldwide [13]. Bangladesh is one of the five countries that were predicted to have the largest number of people with diabetes by 2030 [14]. The lifetime risk for developing DFU could be as high as 25% [15]. Therefore, DFUs may become a huge burden for the country. Patients with DFUs have a 2.5 times higher risk of death at five years than diabetic patients without foot ulcers [16]. Furthermore, in the Indian region, DFUs usually present late at healthcare facilities and frequently lead to amputation of limb(s) [17]. However, proper management of DFUs can greatly reduce, delay, or prevent complications such as infection, gangrene, amputation, and death [18].

Cutaneous wound healing is a complex procedure, and healing of DFUs needs the successful application of several steps, including patient education, debridement, offloading, and appropriate dressing [7,18]. Various advanced dressings are available for DFUs, and collagen dressing is one of them [18].

This comparative study was conducted in 100 patients with DFUs, where 50 patients were treated with a conventional dressing and another 50 patients were treated with a collagen dressing. The mean age of 100 participants was 60.07±10.39 years, and the majority of the patients (42%, n=42) were in the age group of 61-70 years. Additionally, the majority of the patients were found to be male (77%, n=77). This could be explained by the fact that advanced age and male sex are two of the important risk factors for DFUs [18]. In a comparable study, Janmohammadi et al. reported a nearly similar mean age of 58.8±11.23 years, and the prevalent age group in their study was also 56-65 years (38%, n=38) [19].

The majority of the participants were Muslim (96%, n=96). This figure represents the Muslim majority of the Bangladeshi population. In Bangladesh, where most of the population is Muslim, religious and cultural practices, such as ablution, fasting, and traditional healing, can influence health-seeking behavior and wound care. Recording participants’ religious background helps contextualize treatment adherence and acceptance of advanced dressings like collagen. Of the patients, 52% (n=52) came from rural areas, and the rest (48%, n=48) came from urban areas. This is consistent as increasing numbers of people are shifting from rural to urban areas with advancing time. Occupation and rural versus urban residence affect access to DFU care in Bangladesh. Rural patients often face limited access to advanced treatments like collagen dressings, while manual laborers may have difficulty with wound care adherence, impacting healing outcomes. DFUs can be seen in patients coming from a wide range of occupations. However, the ‘other’ category, which consisted of rickshaw pullers, van drivers, farmers, and day laborers, was found to comprise 30% (n=30) of cases. As these jobs can be associated with poor foot hygiene, the higher number of patients coming from these categories of jobs is explainable.

In the present study, 92% (n=92) of subjects had type II diabetes and 8% (n=8) had type I diabetes. Worldwide, type II diabetes is the most prevalent type of diabetes [14]. However, both type 1 and type 2 diabetics are prone to developing foot ulcers [20].

In addition to advancing age and male sex, poor glycemic control evidenced by increased glycated hemoglobin (HbA1C) level, diabetic peripheral neuropathy, and peripheral vascular diseases are some of the important risk factors for developing foot ulcers in diabetic patients [18]. In this study, an overall high average HbA1C level was found (8.09±0.96) in all patients with DFUs. In addition, diabetic neuropathy was found in 78% (n=78) of patients. Regular screening for loss of protective sensation, which occurs due to neuropathy, is an important preventive measure that should be undertaken for the prevention of foot ulcers in this group of patients [21]. 

Prevalence of peripheral artery disease in diabetic patients can be as high as 29% over the age of 50 years, and it is associated with complications like DFUs and critical limb ischemia [22]. In the present study, arterial insufficiency was found in 7% (n=7) of the DFU patients.

Screening for loss of protective sensation, educating patients about proper foot care, periodic foot examinations, optimizing glycemic control, cessation of smoking, intensive podiatric care, debridement of calluses, and certain types of prophylactic foot surgery are some important interventions that can be employed to prevent foot ulcers in diabetic patients [21].

In this study, the duration of ulcer ranged from one to 72 months (median four months) and one to 82 months (median four months) for the collagen dressing group and the conventional dressing group, respectively. Mean wound area of the collagen dressing group and the conventional dressing group were 5.91 cm^2^ (range, 0.57-17.80) and 5.15 cm^2^ (range 0.40-18.40), respectively. In a comparable study, Veves et al. found median wound duration with range for the collagen (Promogran) group and the control (conventional) group to be three months (range, 1-84 months) and three months (range, 1-144 months), respectively [6]. They also reported a mean wound area of 2.5 cm^2^ (range, 0.2-27.4) and 3.1 cm^2^ (range, 0.1-42.4), respectively, for the collagen group and the conventional group, which was lower than that found in this study.

The majority of the ulcers were in the plantar surface of the foot (73%, n=73), and 70% (n=70) had serous discharge. In an epidemiological study, Janmohammadi et al. reported 46% (n=207) of foot ulcers were located under the plantar surface of the foot in the sole and heel [19]. They also reported 54% (n=243) of discharging wounds [19]. Wounds with lesser duration and on the plantar surface of the foot are more likely to come with infection, contamination, and discharge than older wounds and wounds in other areas of the foot. This could explain the high proportion of wounds in the sole and heel with serous discharge in this study.

In normal, uncomplicated cutaneous wounds, granulation tissue usually starts to appear approximately four days after injury, and healing by wound contraction starts within the first three weeks after injury [7]. However, diabetes is an important risk factor that delays wound healing [23]. An appropriate dressing is the mainstay of treatment in DFU[18].

In this study, 50 patients were treated with a collagen dressing, and another 50 patients were treated with a conventional dressing. In the collagen dressing group, granulation occurred in significantly shorter duration (2.26±1.58 weeks) in comparison to the conventional dressing group (3.76±1.57 weeks, p <0.001). Healing time was significantly lower in the collagen dressing group (4.90±2.54 weeks) than in the conventional dressing group (6.24±3.76 weeks, p<0.05), implying that collagen dressing was better than conventional dressing in the healing process of ulcers. Also, the collagen dressing group was found to be more likely than the conventional group to develop granulation tissue (HR 1.96, 95%CI 1.31-2.96, p <0.05) and healing (HR 1.46, 95%CI 1.31-2.96, p>0.05). Several other studies have also shown the supremacy of collagen dressing over conventional dressing in diabetic and non-diabetic ulcer healing [6,11,24].

The study by Veve et al. compared collagen dressing with conventional dressing in 276 patients [6]. Collagen dressing showed borderline significance in superiority of healing over conventional dressing in the subgroup of patients in whom wounds were less than six months in duration. Rao et al. included 75 patients in a similar study and found that healing time, duration of antibiotic therapy, and mean follow-up period were significantly less in the collagen dressing group [11]. A study by Thekdi et al. conducted in India included 100 patients with ulcers who were categorized into two groups; one group received conventional dressing material, and another group received several newer dressing materials, including collagen [24]. They concluded that newer dressing material was superior compared to conventional dressing across four parameters: appearance of granulation tissue, absence of purulent discharge, removal of slough, and healing stage achieved.

Appropriate dressing is the mainstay of treatment in DFUs, and the aim of the dressing is to confer moisture balance, protease sequestration, growth factor stimulation, antimicrobial activity, oxygen permeability, and the capacity to promote autolytic debridement that facilitates the production of granulation tissues and the re-epithelialization process [18]. Collagen dressing is a better dressing for DFU in this regard. Beyond inhibiting proteases, collagen dressings provide a moist, biocompatible scaffold that supports cell growth and angiogenesis. Their antimicrobial properties and ability to promote granulation contribute to faster healing compared to conventional dressings [5,7,8].

Limitations of this study include it being a single-centre study. Furthermore, all factors that can influence the healing process were not considered, and an objective assessment of the severity of foot ulcers was not performed. A long-term follow-up of both groups was not done either, and the sample size was not representative to generalization of the general population. To validate these findings, we recommend a randomized controlled trial with a larger sample size.

## Conclusions

DFUs often require extensive healing time and are associated with increased risk of infections and other sequelae that can result in severe and costly outcomes. In diabetic individuals, it is one of the important causes of mortality, morbidities, and hospital admissions. As different methods are available, the study was done to assess the comparative outcome between conventional and collagen dressings. In conclusion, it was seen that collagen dressing is better than conventional dressing, as evidenced by earlier appearance of granulation tissue and shorter healing time. However, these statistics should be used with caution, as results from this study with a small sample size may not reflect the original picture of this patient group.

## Figures and Tables

**Figure 4:**
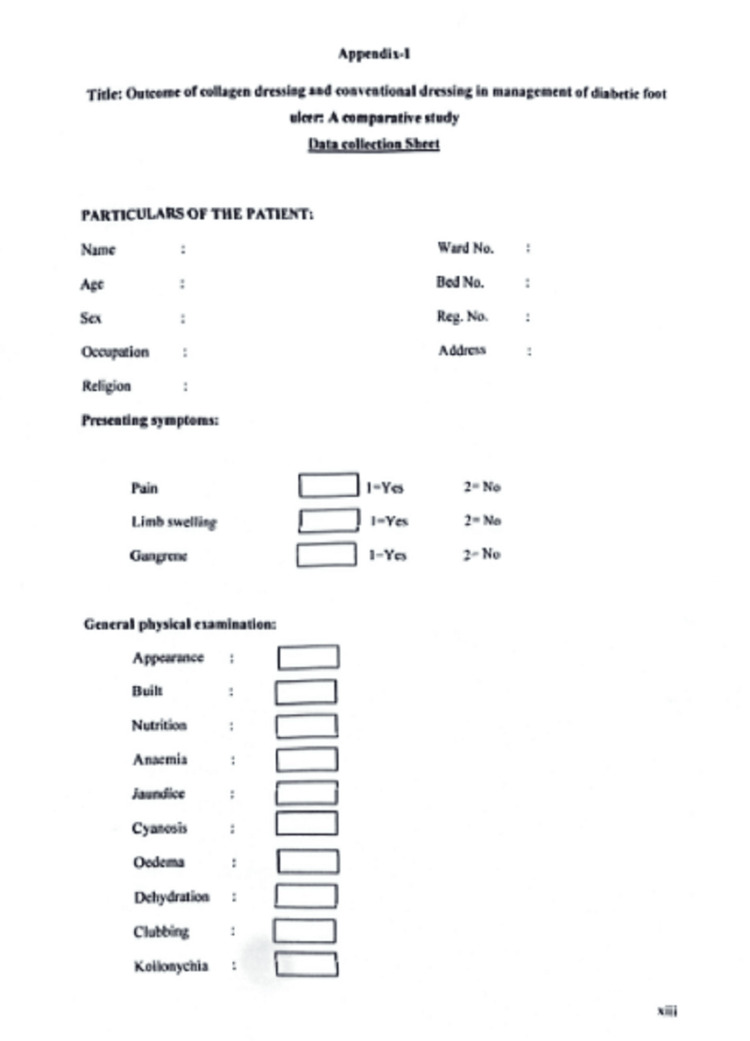
Questionnaire Page 1

**Figure 5:**
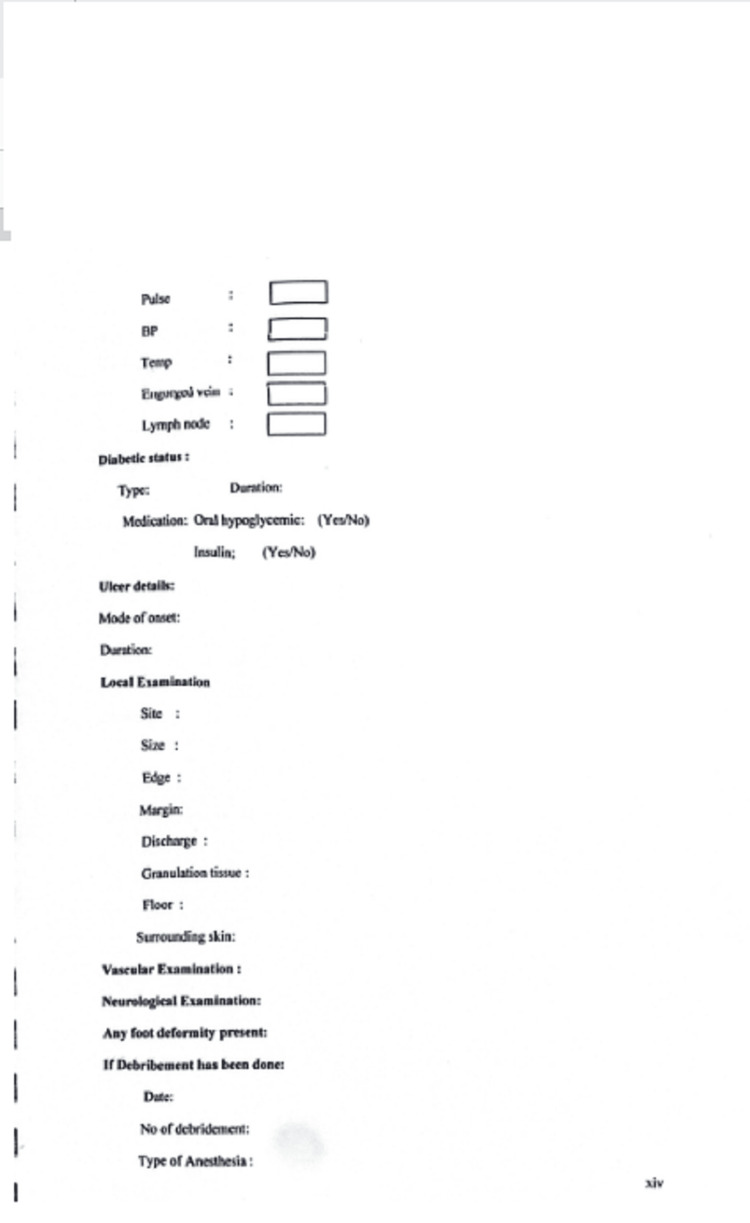
Questionnaire Page 2

**Figure 6:**
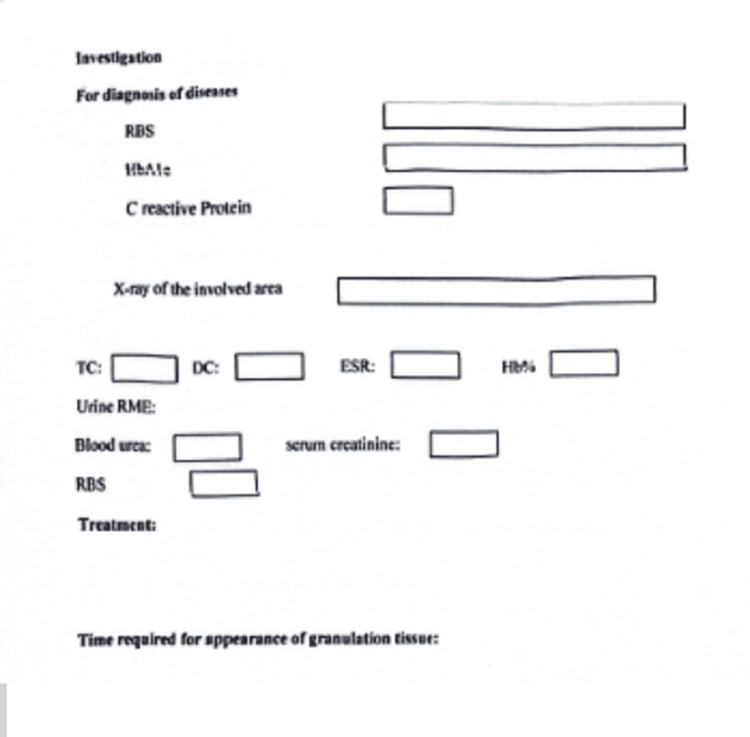
Questionnaire Page 3

## Appendices

Study questionnaire
